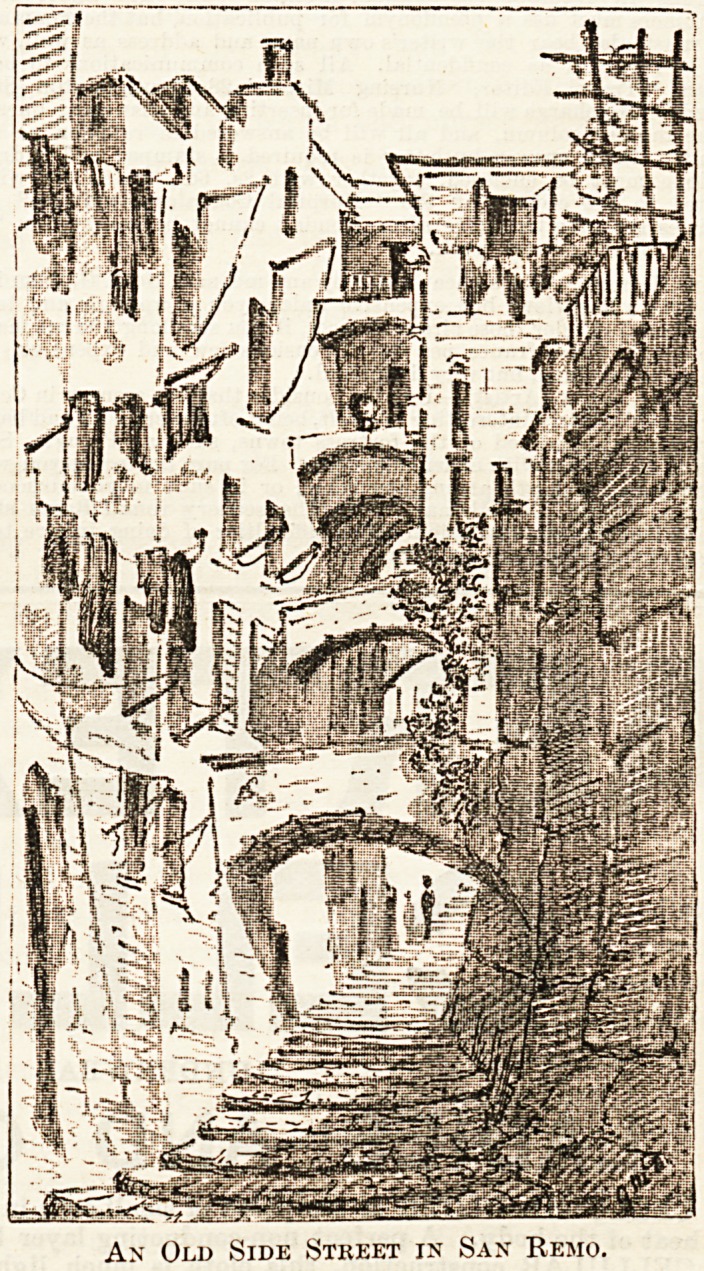# "The Hospital" Nursing Mirror

**Published:** 1899-06-17

**Authors:** 


"The Hospital" Nursing Mirror.

Being the Nursing Section of "The Hospital."

[Contributions for this Section of "The Hospital" should be addressed to the Editor, The Hospital, 28 & 29, Southampton Street, Strand, London, W.C., and should have the word "Nursing" plainly written in left-hand top corner of the envelope.]

### Notes on News from the Nursing World.

### THE REPLY OF PRINCESS CHRISTIAN TO HER CRITICS.

At the annual meeting of the Royal British Nurses' Association on Saturday the Princess Christian made a brief but effective reply to the offensive representation that in her efforts on behalf of the organisation she has held up nurses and the nursing profession as objects of charity. "I am very sorry," said Her Royal Highness, "for the people who made that remark." It gives point to this dignified rebuke that the Duchess of Connaught has consented to become a vice-president of the Association. The Princess Christian was able to announce officially that, as intimated in these columns on May 20th, the sum realised by the Café Chantant exceeded £1,500. In these circumstances, and in view of the satisfactory nature of the report, the members of the association are not likely to be disconcerted by carping comments on the part of persons who have vainly endeavoured to discredit the movement.

### THE PULPIT AND THE NURSING PROFESSION.

Several of the preachers on behalf of the Hospital Sunday Fund alluded, in eloquent language, to the ministrations of the nurses at the great charitable institutions of London. The Rev. J. Hasloch Potter, vicar of Holy Trinity, Upper Tooting, and rural dean, referred on Sunday evening to the elevation of the nursing profession, and said it was a matter for congratulation that the work of nursing was now discharged by cultured and gentle ladies who had done much to effect a revolution in the treatment of the sick and suffering. He also dwelt upon the value of the experience they gather in the hospitals, which, as he reminded his congregation, are not only of immense benefit to the patients, but, owing to the schools attached to them, are invaluable factors in the promotion of medical science and surgical skill. Dr. Cobb, at St. Ethelburga, Bishopsgate Street, also paid a warm tribute to the work being done by doctors and nurses, whom he described as "those priests of a suffering humanity."

### THE RECREATION OF NURSES.

It is curious that both Boards of Guardians and a section of the public should be so slow to realise that nurses need recreation even more than the majority of workers because of their long hours and close confinement. At Yarmouth the other day objections were raised to the introduction of a piano at the workhouse; and there has been quite an outcry at Camberwell because the plans for the new Havil Street Infirmary include tennis courts for the nurses. The provision, instead of inciting censure, deserves commendation. As one of the crowd said at an "indignation" meeting, "Why not? Don't the nurses work hard enough to entitle them to a little recreation?" Another point is that unless nurses have adequate recreation it is absurd to expect that they can keep the bright faces and maintain the cheerful manner which are so keenly appreciated by the patients.

### HOW NOT TO EMPLOY SPARE TIME.

The clerk to the City and County of Bristol Board of Guardians sends us a letter asking us to make known the fact that a trading firm in London has been communicating with nurses in workhouse and other hospitals endeavouring to secure a fee for registering them in the books of the company, for the receipt of patterns, materials, &c., to be made up by nurses in "spare time." The communication states that the company "have many nurses and other poor law officers who, when on either day or night duty, without the least neglect of their duties, sit and pass away in profitable and easy employment many a pleasant hour that otherwise would be dull and tedious, while at holiday time, &c., the few extra pounds in hand prove very acceptable." But the Bristol guardians, while not thinking it likely that many poor law officers will forward the required registration fee, consider "it most improper that officers should be canvassed with a view to the employment of a portion of their time on behalf of a trading firm." Their protest is quite natural and proper, and we might have more to say on the subject if the clerk, in his postscript, had not stated that a letter addressed to the company in question has just been returned through the post, marked "gone away."

### THE INTERESTS OF WOMEN IN PARLIAMENT.

Since the measure securing seats for shop assistants in England and Ireland has passed the House of Commons without opposition, it is believed that Lord Salisbury will not persist in his hostility to the proposed legislation. Of course, if the Bill is placed on the statute book, it will be extended to Scotland. . . . Women must not yet assume that they will be able to serve as aldermen and councillors upon the new borough councils to be created under the London Government Bill. The measure has to run the gauntlet of the House of Lords; and the House of Commons is so evenly divided on the question that the Peers, spurred on by the Chancellor of the Exchequer, are as likely as not to strike out Mr. Courtney's amendment. While we see no particular objection to the advent of a few alderwomen and lady councillors, such statements as that of Sir Arthur Arnold, who thinks that the office of Archbishop of Canterbury should be open to women, are distinctly calculated to excite prejudice and distrust.

### NURSING IN THE TROPICS.

Last week Major Ronald Ross, lecturer in connection with the Liverpool School of Tropical Diseases, delivered an address to the nurses at the Royal Southern Hospital on "Nursing in the Tropics." Dr. John Cameron presided, and there was a good attendance, the company including Mr. William Adamson, the president of the institution. Major Ross, in the course of his lecture, pointed out that nursing in the tropics was essentially very different from nursing in this country. In the tropics they had quite a separate set of diseases to deal with, of which they could have no experience here. Then they could not know anything about the hospitals in the tropics; neither could they be acquainted with the manner of nursing the natives; and lastly, and perhaps most important of all, they did not know how to keep themselves in health abroad. On all these points it was desirable that nurses should have the fullest information, and the new tropical school founded in Liverpool would endeavour to impart it. So far as the tropics were concerned, the subject of nursing had been very much neglected; in fact, there were many hospitals in India, for instance, with no nurses at all. The lecturer touched on the difficulty of dealing with native patients, pointing out that much trouble was apt to arise owing to caste prejudice. He also imparted valuable information to the nurses on the subject of preserving their personal health in tropical climates.

### COMPLIMENTS FROM DROGHEDA.

Striking testimony was paid at the annual meeting of the Drogheda District Nursing Association to the work of the nurses. Dr. Parr, following Father Clarke, who warmly eulogised their efforts, said that "no one, except clergymen and doctors, and perhaps the ladies of the committee, could have any idea of the great benefit that the nurses were to the sick poor. When they saw squalor and sickness in poor cabins, and often the poor mother lying in filth with her children around her, the doctors invited one of the nurses to pay a visit, and when they went next day it was wonderful to see the transformation. They saw a smile where they previously saw tears, and many a time he had heard blessings poured upon the heads of the nurses by the poor patients." It may be added that the nurses at Drogheda work for eleven hours per day every day, including Sundays.

### NURSES AND THE WORKHOUSE PORTER.

According to the *Western Daily Press* 15 nurses at the Eastville Workhouse sent in their resignations to the Bristol Board of Guardians because they objected "to report their outgoings and incomings to the porter at the lodge of the institution." The resignations were at first accepted, but on reconsideration it was decided to refer the question to the Local Government Inspector, with the view of the adoption of a more conciliatory course. It is clearly necessary that a record should be kept of the hours at which the officials and servants of an institution pass the gates, but the person to whom this duty is confided should, of course, be a discreet and trustworthy individual.

### THE TWO MILLION SURPLUS WOMEN.

At the annual meeting of the Society for the Employment of Women on Tuesday, Sir Owen Roberts, who presided, said that in England there are two million women in excess of the male population; while, in the Colonies, the surplus of males is about the same. It would be an easy solution of the problem of employment for the former if the balance could be redressed by marrying the surplus women at home to the surplus men in the colonies. But, apart from the fact that marriage is by no means the goal of every woman's ambition, this mode of dealing with it is not practicable. Meanwhile, Lady Knightley, who moved the adoption of the fortieth report of the society, showing that in the past year 49 women had obtained permanent engagements, and 248 temporary work through its agency, suggests that her sex should turn their attention to dairying and in the direction of laundries. To a certain extent they have already done so, but there is yet some room in these markets for their talents.

### A THEATRE NEXT DOOR TO A HOSPITAL.

It is not surprising that the General Purposes Committee of the Metropolitan Asylums Board object to the scheme for erecting a theatre and opera-house on a site immediately adjoining the North-Western Fever Hospital at Haverstock Hill. The opposition is based upon the contention that a theatre in such close proximity would be most "undesirable in the interest of the patients." Some people may be of opinion that it is not desirable in the interest of the public. But the public can take care of themselves. If, however, the noise of the performance is likely to interfere with the patients, retarding their progress, in spite of the most careful nursing, the London County Council will be justified in refusing consent to the plans for the new building.

### SHORT ITEMS.

The Duchess of York has consented to open the new nurses' home of Queen Charlotte's Hospital, Marylebone Road, on July 17th.—The Duchess of Portland, wife of the president of the British Lying-in Hospital, visited the institution on Friday last. Her Grace, who was conducted over the building by the matron, Miss Mabel Cook, evinced much pleasure in what she saw, and expressed her satisfaction with the arrangements. The original minutes and cash book of 1749, in which the names of the Dukes of Portland first appear, were produced for the inspection of the Duchess, and the visitors' book, instituted in the 150th year of the hospital's work, was signed.—The friends and subscribers to the nurses' home at Plaistow assembled in force on Monday afternoon, when the Countess of Selborne and Lady Ebury were "At Home." A notice of the work appeared in a recent number of the *Nursing Mirror*.—Last week Miss Healley had an "At Home" at her new nursing home in Fulham Palace Road.—Continued progress is reported by the Derby and Derbyshire Nursing Association. The staff now numbers 68 nurses, of whom 45 are engaged in private nursing, eight in district nursing, and 15 are probationers. Last year they earned £2,655. We hope that the proposal to establish a sanatorium in connection with the nursing institute in the neighbourhood of Derby will meet with the support it merits.—The Barton Board of Guardians is in the unenviable position of having the smallest staff of nurses in proportion to the inmates of any workhouse hospital in Lancashire —namely, that of one to 107. The Poor Law inspector also states that he found 14 pauper assistants!—On Friday, June 23rd, a garden fête and sale will be held at the Hospital and Home for Incurable Children, 2, Maida Vale. The sale is under the patronage of H.R.H. the Duchess of Connaught, and will be opened at three p.m. by Lady Grant Duff. It is in aid of the hospital, and will be prettily arranged in the garden of the institution. The boys' band of the Foundling Hospital will play during the afternoon, and there will be short entertainments at intervals indoors.—Mr. and Mrs. Martin, the son-in-law and daughter of the late Henry Lewis Raphael, have endowed a cot in Guy's Hospital as a memorial to him.

### Gynæcological Nursing.

By G. A. Hawkins-Ambler, F.R.C.S., Surgeon to the Samaritan Free Hospital for Women; Assistant Surgeon to the Stanley Hospital, Liverpool.

### THE QUALIFICATIONS OF THE GYNÆCOLOGICAL NURSE.

A good gynæcological nurse must first be a good general nurse. In nursing, as in anything else, specialism is apt to be bad, or only indifferently good, unless it naturally develops out of special tastes, proclivities, and success discovered in the course of general training and experience. So the well-trained special nurse will be an excellent all-round nurse whose special ability has been recognised, whose taste in a particular direction has naturally grown, or who has chanced on special work in the course of her professional duty.

All this argues, of course, that she will be older, not younger, than the average nurse. I do not think any woman under twenty-three should take up nursing or undergo the moral and physical strain of the profession at an immature age. But the nurse who takes up gynæcology for any reason should be to a certain extent seasoned, balanced, fitted by a graver and more serious temper for nursing a class of cases in whom we find not only physical and surgical conditions of extreme gravity that entail an enormous strain on all their attendants, but many patients whose moral disposition is light and who have at times too little respect for the ethics and responsibilities of sex to make it good for any woman who is too young, inexperienced, or ill-balanced to be brought into close contact with them.

Given age, physical strength, experience, and a judicious mixture of sense and sensibility, the gynæcologist would ask little more of his nurse unless it were a conscientious and unswerving obedience to orders, a particular respect for detail, and a courageous readiness to do what she has been taught to do at the time it is called for, without delay or equivocation. Emergencies arise in gynæcological nursing that have to be met on well defined lines, and with promptitude and resourcefulness. This is a very different thing from mere meddlesomeness. I once had to employ two new nurses for a hysterectomy, and met them on the stairs the day after the operation carrying castor oil with which they were about to dose the patient, for no earthly reason except that another surgeon had given an aperient under some special circumstances. Without asking a question, or stopping to consider that even if the treatment had been routine treatment it might under certain conditions have ruined an operation or destroyed a patient, they rushed into a perfectly unjustifiable proceeding. This was an entirely different thing from my own nurses' action in giving a turpentine enema or an aperient after an abdominal section, on the appearance of distension, without waiting for orders; though in no case would treatment be initiated if the surgeon were within reach to be consulted.

But these are obvious propositions, and I need not argue them to readers who are trained nurses. What we need to insist on in the nurse who attends a gynæcological case are a steady hand, quiet manner, cheerful and willing service, and the possession of sympathy that does not degenerate into disquieting sensibility.

Indeed, what is required in a perfect nurse is a magnificent sympathy, which shall make her understand her patient completely, and stand between her and anything that will tend to aggravate her sufferings, to irritate her, or cause needless pain. The patient's consciousness of this sympathy has a wonderful effect in minimising that querulousness of disposition and chafing under affliction which intensifies physical suffering and delays the progress of cure. The nurse, too, should possess tact and adaptability to enable her to give the proper direction to sympathy, in the way of helping where she can help, and not interfering with needless fussiness where a patient is best left alone. For patients are often hysterical—they take perverted views of life; the duration of their symptoms, and the gossip of friends, nurses, and doctors have all tended to fix their minds so much on their physical ailment that it has become magnified by the concentration of their ideas into something quite disproportionate to the actual condition. On cases like this the good nurse will have the effect of a moral tonic, will be cheerful and bracing. Her firm, unhurried demeanour gives evidence of knowledge and skill on which the patient can rely, and the impression of a reserve of strength on her part will do more to calm and soothe a patient than any other quality she can possess. Sometimes she will make light of a patient's sufferings, and the consciousness of her sympathy will make the patient take this in a proper way; while at other times, when it is impossible to hide from a woman that her condition is grave, if she knows that her attendants understand why it is grave and are prepared with means to relieve or cure it, she will the more readily fall into the state of mind which is the best basis for a cure.

If the discipline of the sick-room be maintained there will be a mutual understanding between the nurse and the doctor, which does not mean, by any means, familiarity, and which can exist even when these two functionaries have an unfortunate personal antipathy. If they have any good sense they will sink personal dislikes for the sake of their patient and in the interests of science.

Proper reports will be made at regular intervals of the patient's condition. The temperature will be taken at the time specified by the doctor. Medicines, applications, nutrient enemata will be administered with due regularity, every incident bearing on the patient's condition will be noted and described for the doctor, who will give it its proper perspective, and it will be the nurse's duty to make her report as nearly as possible a picture of the patient between the visits of the doctor, so that he can cover the period between them by a lucid and intelligent description of the events that have intervened. This, combined with trustworthiness in details, unquestioning but interested obedience to orders, and a tactful management of the patient, will make a nurse the acquisition she ought to be to both doctor and patient.

I have already referred to the danger of gossip. It amuses a patient probably, and it is very easy to fall into the habit of thus beguiling time, but you must also reflect that it loses you your patient's confidence. If you gossip about other people you will also gossip about her, and sooner or later she will remember that to your discredit.

And if you talk to her much about her case you do not merely increase the harmful concentration of her ideas on herself, but you run the risk of criticising the doctor and his treatment in permitting your personal likes and dislikes to come between you and your duty, which is, to maintain the confidence between doctor and patient—an essential condition of successful practice.

(*To be continued.*)

### To Nurses.

In order to increase and vary the interest in the *Mirror*, we invite contributions from any of our readers in the form of either an article, a paragraph, or information, and will pay a minimum of 5s. for each contribution. All payments are made at the beginning of each quarter, *i.e.*, January 1st, April 1st, July 1st, and October 1st.

### The Nursing of Phthisis in Sanatoria under the "Open-Air" System.

By the MATRON of the National Sanatorium for Consumption, Bournemouth.

### IV.—SPECIAL FEATURES OF A NURSE'S DUTY IN THE OPEN AIR.

WE now come to the consideration of the nurse's duties regarding the actual open-air treatment of the phthisical. In the first place we must realise that the object of this treatment is to cure the lungs in a perfectly rational manner by giving them all the rest possible under the very best hygienic conditions; increasing and maintaining the bodily strength of the patient while feeding his lungs with pure air, and as far as possible reducing the risk of fresh infection, both to himself and the rest of the community, by removing him from crowded and unhealthy neighbourhoods to a locality suitable for his condition.

"Open-air treatment," then, may be described as consisting chiefly of a carefully-arranged diet and an outdoor life, the prominent features of which are rest in a reclining position, and, as the lungs heal, carefully graduated physical exercises under medical supervision.

The question of diet has already been discussed, and, under the head of ventilation, the regulation of fresh air in the sleeping wards during the night. There remains, then, the nurse's duty in the daytime to consider, and this can best be done by first taking a brief glance at what we may term the mechanical part of the treatment.

In the grounds of this sanatorium two "shelters" have already been erected for the reception of patients when the wind is too keen or the weather too bad to allow them to lie unprotected in the open air. These shelters face the south; they are oblong structures having their south and west sides composed entirely of enormous doors, with a hinge in the middle of each. The north and east sides consist of wooden walls to about the height of an ordinary dado. Above that height the east side is formed of two very large, broad French windows, opening outwards, whilst the north side consists of six large windows of the usual fanlight pattern, turning on a pivot at the centre. At the back of each shelter on the north side (interior) a flap table is being constructed to permit of suitable cases taking their meals in the open air.

It will be seen at once that this arrangement of doors and windows, allowing the closing of that side of the shelter which faces the wind, enables patients to lie out practically in the open securely sheltered from the blast and getting the full benefit of the sunshine. The floors of the shelters are stained and oiled; the roofs are one tiled and the other of thatch. They are situated in the highest part of the grounds on a well-drained terrace, which is much used by the patients when there is no wind. As "rest" forms an important part of the treatment plenty of lounge chairs, with foot-rests attached, are provided (these can be obtained from any furniture dealer at a cost of about 12s. 6d. each), and the patients are instructed to recline on them, comfortably wrapped in shawls and rugs, with hot bottles, if they wish, for their feet. The nurse will find that the most convenient hot-water bottle for the purpose is the tin cylinder, 10 in. high and 4 in. diameter, it being fitted with a ring at the top to carry it by. Care must be taken to fill the bottles quite full, otherwise when they cool atmospheric pressure will cause them to leak. The amount of time to be spent by each patient in the open-air is carefully regulated by the physician. The patient's temperature taken night and morning, and when necessary more frequently, is a useful indication of "fitness for treatment"; thus, as a general rule, patients with a morning temperature of 100 deg. and over are kept in bed under open windows until it goes down to normal.

One marked result of the open-air treatment of consumption lies in this reduction of temperature; even in very advanced and hopeless cases it seems to add much to the general comfort of the patient.

Our physicians place the patients undergoing the open-air treatment in two classes—(1) those who are fit to undergo the full treatment; (2) those whom they consider should only be in the open air for a limited number of hours each day. Full open-air treatment means at least eight hours out of the twenty-four at the present time of year in the open air, viz., 9.30 a.m. to 12.15 p.m., 1 p.m. to 4.45 p.m., and 5.30 to 7.30 p.m. As the summer advances an additional hour after supper or the meal hours out-of-doors are prescribed.

The partial treatment consists of from three to five hours spent in the fresh air daily, according to the wind and weather. Certain of the patients are allowed drives in the country-side, and sea excursions are strongly advised in the later steps of the treatment. The resident medical officer also carefully prescribes each morning the amount of physical exercise he considers advisable for each patient. These are a kind of elementary calisthenics very like the first drilling exercises of children, and it is the nurse's duty to see that they are carried out and not prolonged beyond the allotted time. Their main object is to develop the chest, to prevent the tendency of the shoulders to become rounded as the lungs heal and contract, and to train the patients to breathe deeply and through the nose. Nothing is done until all signs of active disease in the chest have ceased and the temperature has become normal in the evenings, in order to avoid any risk of pulmonary hæmorrhage. The exercises are under no circumstances extended beyond a few minutes.

At present this is not what is technically termed a "closed sanatorium;" those patients for whom the doctor considers longer walks are desirable than can be obtained within the limits of the grounds are permitted to stroll in the public gardens adjoining the sanatorium. Before leaving the institution each patient is presented with a copy of a leaflet which has been drawn up pointing out in simple words the common sources of the infection of tuberculous disease and the precautions that consumptive persons must take, not only to prolong their own lives, but also to prevent them from becoming fresh centres of infection to those around them.

In an earlier paper I spoke of the education of patients in points of personal hygiene as being included among the nurse's duties. She must impress upon those in her care the great importance of personal cleanliness in such details as always washing the hands before meals, lest they re-infect their own systems, and for the same reason teach them to keep their nails as clean and short as possible. They should bathe at least three times a week—oftener if possible. Male patients especially must be exhorted to keep the hair on their faces spotlessly clean; theoretically, of course, they should be clean shaven. If possible a consumptive patient should sleep in a room alone, and always in a separate bed. He should never kiss or be kissed by anyone. After the death of a phthisical patient his personal clothing should be disinfected or burnt, and (in private houses) the room disinfected thoroughly, together with its contents, the wall paper being stripped off before another is put on.

In closing this short account of nursing the phthisical I would wish once more, at the risk of repetition, to impress upon all nurses in sanatoria what an immense responsibility rests upon their shoulders, and what an important part they play as what may be termed "popular exponents" of the hygiene of the Nation.

### Cases of Interest.

### CHEYNE-STOKES RESPIRATION.

A correspondent writes: I am just now nursing an old lady suffering with carcinoma. As I entered the room a week ago the rather heavy breathing stopped; I was startled and thought I had come too late to help care for her. One of the daughters noticing my listening attitude said she had breathed like that ever since she had had morphia. It continued most of the night. Six or seven breaths, and then perfect silence and stillness, except for the fluttering heart, for fifteen seconds. The second night there was perfect silence, but a slight rise and fall of the chest and abdomen. The third night thirteen or fourteen breaths, then neither sound or movement for thirty-five seconds; that continued for several hours. When the patient is awake the breathing is normal, but though no morphia has been given for two or three days when asleep the breathing is as mentioned, though not quite as regular as when first noticed. The breathing commences quietly, gaining in strength, then dying away again, then perfect silence and stillness for from fifteen to forty seconds. I should be glad to hear through the columns of your paper if this is a common occurrence.

*** The above is a very good description of what is known as Cheyne-Stokes respiration. It occurs in many conditions, and probably has to do with disturbed nutrition of the respiratory nervous centre. Often the respiration becomes quite noisy when it is at its deepest, which makes the silence of the interval still more striking. Probably if our correspondent laid her hand on the upper part of the abdomen on the bare skin she would find that some slight diaphragmatic respiration goes on during part at least of the period of apparent cessation of breathing.—Ed. *T. H.*

### Minor Appointments.

Miss Annie J. Hobbs has been appointed Night Sister at the Hospital for Women, Soho Square. She received her training at the West London Hospital, where she remained on as staff nurse in the wards and nurse in charge of the out-patient and casualty departments respectively. Since January, 1898, she has had charge of a floor at the establishment for Invalid Gentlewomen, 90, Harley Street.

The Cottage Hospital, Bourton-on-Water.—On June 3rd Miss Isobel C. Sherlock was appointed Matron. She was trained at the Children's Hospital, Liverpool, and at the Leicester Infirmary, and has since been nurse at the West Kent Hospital, Maidstone, and at the Halstead Cottage Hospital.

Maidenhead Union.—Miss Alice Wilson has been appointed Assistant Nurse at the above union, and will commence her duties on the 17th of this month. She was trained at St. Peter's Home, Woking, by the Meath Workhouse Attendants' Association.

Royal Victoria Hospital, Bournemouth.—Miss Agatha L. Laughlin was elected Charge Nurse last month. She was trained at the Royal Free Hospital, Gray's Inn Road, and subsequently held the appointment of probationer at Worthing and Willesden.

Isolation Hospital, Gorse Hill, Swindon.—Miss Janet Dundas was appointed Charge Nurse on June 5th. She was trained at the Belvedere Hospital, Glasgow, and has been head nurse of the Ophthalmic Institution, Glasgow, and charge nurse of the Public Health Hospital, Leith.

Horton Hospital, Bunbury.—Miss Alice Holroyd was appointed Charge Nurse of wards and operating theatre May 23rd. She was trained for three years in medical and surgical nursing at Ancoats Hospital, Manchester.

Royal Hospital for Sick Children, Edinburgh.—Miss Jean M. Wright, the new Assistant Matron, was for *two* not *ten* years sister at the Grimsby and District Hospital.

### Annual Meeting of the Association for Promoting the Compulsory Registration of Midwives.

Lady Balfour of Burleigh presided over the annual meeting of this association, which was held last week at 55, Cadogan Square. The room was well filled.

Mr. Heywood Johnstone, M.P., moved the adoption of the report, and in the course of his remarks he reviewed the Parliamentary position of the Bill now before the House of Commons. They had, he stated, been unfortunate in the ballot, and had only secured twenty-five minutes' discussion before the measure was shelved for the session. Nevertheless during that short debate the Bill had been warmly commended by Sir William Priestley. "The public position," he continued, "was very much better now than last year. Some freezingly cold water had been thrown upon the deputation to the Duke of Devonshire, but it had been accompanied by excellent advice and valuable suggestions. The association had been recommended by his Grace to confer with the General Medical Council and to frame a measure acceptable to the medical men on the committee. The result of acting upon this recommendation was that the Bill, as now drafted, meets with no serious objection from the medical profession, and has received the approval of the Royal College of Physicians and of the County Council Associations." Mr. Johnstone urged his hearers to drop details, which could be adjusted afterwards, and to fight for the principles of the measure. The first vital principle was that habitual midwives should not be permitted to pursue their calling unless they were qualified to do so; and the second, that all midwives should be under local supervision. He believed that the Bill ensured this in the best possible way by placing the licensing to practise in the hands of the County Councils. It was not only a protection against crime, but it secured sanitary precautions which could not be too early enforced for the welfare of the public health. Mr. Johnstone concluded by thanking Dr. Cullingworth for his pamphlet, and the friends who had brought the topic up for discussion at the National Union of British Workers at their last conference at Norwich.

Mr. Roumieu, in seconding the adoption of the report, spoke of the urgent necessity of something being done to remedy the existing state of things from a coroner's point of view.

Dr. Rice cited some graphic instances from his own experience of the state of affairs in Derby during the past twenty years. When he received his first appointment, because he had taken honours in midwifery, the Guardians were unanimously of opinion that "something must be done," though no one knew exactly what to do. "Sairy Gamp," he said, "was a very estimable person compared to the women who stuck a card with 'midwife' on it in their windows." In the thickly populated slums of Derby four only knew anything of their work, the rest were awful specimens. One laid out a child that had died of virulent scarlet fever in the middle of a long case, with the result that ten women lost their lives and three became chronic sufferers. Another nursed her husband through an attack of erysipelas and continued her maternity work at the same time. He had distributed short leaflets with simple directions by the shoal, and he read one to a so-called midwife, as she could not read herself. She, however, disagreed with everything in it, "as she had had 14 children and never treated any of them in such a manner." Dr. Rice asked, "How did you rear them?" and she replied, triumphantly, "Oh, three lived!"

The executive officers having been re-elected, and the honorary treasurer having pleaded for more liberal support during the coming year, the meeting terminated.

### The History and Development of Trained Nursing.

### II.—THE PROGRESS OF NURSING.

We have seen how the great movement of nursing reform was beginning, from 1840 onwards, to make itself felt in England. It has been somewhere said that the men and women who sway the world in these latter days are not so much the originators of a brilliant thought as those to whom it is given to focus and voice unuttered feelings already stirring amongst a people. To Florence Nightingale, in the history of trained nursing, belongs this honour. The publication of her "Notes on Hospitals," containing the essence of those principles of hospital construction and management and the care of the sick, now of universal acceptance, but which then came as a new gospel, aroused general attention; to its author the Government naturally turned at the Crimean crisis. How nobly Miss Nightingale responded to the call is matter of common knowledge, and need not be entered into here. The foundation of the Nightingale School of Training for Nurses with the money subscribed by a grateful people to the woman they delighted to honour really marks the introduction of system into the work of nursing, and the birth of the nursing profession in Anglo-Saxon countries. When trained women were needed to go out to the Crimea, ten Roman Catholic nuns, eight Anglican Sisters of Mercy, and one other lady were all that were available; the rest following as quickly as they could be put through a hurried course of instruction. And the only instruction attainable was extremely insufficient, for the difficulties in the way were great indeed. Educated women had not thought of sick nursing as a profession, and the hospitals were, Miss Nightingale wrote in 1851, schools, "it may almost be said, for immorality and impropriety, inevitable where women of bad character are admitted as nurses."

In 1856, Mrs. Wardroper was appointed matron of St. Thomas's Hospital, and had effected such improvements that, after investigating the conditions of nursing at the chief English hospitals, Miss Nightingale expressed her desire to establish her training school there, placing its direction in the hands of Mrs. Wardroper. In June, 1860, the Nightingale School was opened, with fifteen "probationer nurses." In this year Miss Nightingale brought out her "Notes on Nursing," which in spite of the lapse of time and the progress of the art of nursing, yet contains almost all there is to be said of vital importance on the subject.

Meantime one hospital after another was reforming its ways. The sisters of St. John's House took over the nursing of King's College Hospital, Charing Cross Hospital, the Children's Hospital at Nottingham (besides the Galignani Hospital in Paris), whilst University College Hospital placed its wards in the hands of the All Saints' sisters. St. John's House was now sending out private nurses and giving a year's training to a large number of women in the various institutions under its control.

Provincial hospitals soon began to follow suit, appointing as matrons women trained in the London hospitals, and starting upon the work of training probationers for themselves. In 1865 Agnes Jones, taking with her twelve nurses from St. Thomas's Hospital, began the work of organisation at the Liverpool Workhouse Infirmary, this being the first introduction of skilled nursing into the Poor Law Service. Constant application was made to the Nightingale School for pioneers to organise schools of nursing throughout the country; district nursing societies sprang up on all sides; the training of monthly nurses was begun systematically at Queen Charlotte's Hospital; Her Majesty's Army and Navy Nursing Sisters took over the management of the naval and military hospitals. Each year saw the establishment of new centres of training—new agencies for carrying on the work of skilled nursing in hospitals and amongst the rich and poor in their own homes. The Queen, ever interested in the progress of nursing, gave her special patronage to district nursing, devoting her Jubilee offerings to the establishment of the Queen Victoria Jubilee Institute.

Nor was the pioneer work confined to the United Kingdom. Let us see how English nurses were spreading their systematised knowledge into other lands.

In 1870 Miss Florence Lees went out to Metz to assist in nursing the wounded in the Franco-Prussian war, subsequently taking charge of the Ambulance Hospital at Homburg, Miss Byron and a staff of sisters going on a similar errand. In 1873 Sister Helen, of King's College Hospital, went out to organise the first modern training school for nurses in the United States of America at the Bellevue Hospital, New York. Two years later nurses went to Zanzibar and to Bucharest, while Miss Machin left her post of home sister at St. Thomas's to organise the General Hospital, Montreal. In 1882 Miss Furhmann, who had been sent by the Crown Prince and Princess of Prussia to the Nightingale School for Training, took charge of the Nurses' Home at Berlin, and later of the City Hospital. Nurses started work in Alexandria and in Rome. Miss Alice Fisher in 1884 went from the Nightingale School to establish trained nursing at the Blockley Hospital, Philadelphia, from whence her pupils spread her teaching all over the country. Miss Crisp went to Auckland and instituted a system of nursing in New Zealand. English nurses were placed over the hospitals in India, Australia, and Ceylon, and the Queen of Sweden appointed a Nightingale nurse to the charge of her training school for nurses at Stockholm.

Less than 50 years had thus seen a complete revolution within hospital walls and the evolution of a new profession for women. The period of training for a nurse gradually lengthened itself from a few months to a year under Miss Nightingale's code of rules, and slowly since then to the three years' term, which is now practically adopted everywhere in the large training schools, in practice even if not in name. Many changes have taken place, not always without friction. Lectures and examinations were instituted, and the schools began to grant certificates to the graduates. The introduction of the new system naturally created difficulties; there were troubles between the sisterhoods and the committees of the hospitals they nursed, resulting for various reasons in the gradual elimination of the religious element from the lay institutions. There was the historic "crisis at Guy's," when the medical staff objected to the somewhat unceremonious upset of old methods. Later came the attempt to introduce State registration, a move strongly opposed by many, who held that it would lower the ideal of nursing. The profession of nursing has grown with almost phenomenal rapidity, and recently, in the haste to assert the "status" of the nurse, there seems some danger lest more important matters should be overlooked. In England to-day the development has been one-sided. While many people are feeling that it is possible for the hospital nurse to become even too highly trained, there is a no less fatal tendency to be content with semi-trained women in rural districts, and it is to the shame of England that in attention to maternity nursing and midwifery we are far behind other countries. There is much, too, to be done in raising the standard of workhouse, fever, and asylum nursing.

In lamenting the present want of cohesion amongst the nurse training schools in England and the absence of any uniform curriculum, to which some people attach a very disproportionate importance, it must be remembered, as Miss C. J. Wood well puts it in an article in the June number of *Nursing Notes* on "The Present Position of the Nursing Profession," that "the growth of the profession is the outcome of individual work carried on in isolated places, that the workers have been so engrossed with the work that the details of organisation have had to wait"; and it may well be that the time is even yet not ripe for the last step to be taken towards the consolidation of the profession as such.

After all, the crying needs of the sick brought the nurse into existence, and the first efforts of the pioneers of trained nursing were directed to the alleviation of suffering, not to the building up of an employment for women. It is rather to the honour of the workers that the former aim has swamped the latter, and if English nurses will but remain true to the standard placed before them by such women as Florence Nightingale and Agnes Jones, they may await with patience the realisation of their professional ideals in the future.

### Echoes from the Outside World.

AN OPEN LETTER TO A HOSPITAL NURSE.

The assumption that the failure of the Conference at Bloemfontein means war in the Transvaal is only that of the hasty—who do not, or will not, see that patriotism demands the pursuance of a policy of patience. The British Government, intent upon avoiding, if possible, a calamity which would inflict untold misery upon thousands of our fellow countrymen, their wives, and families, in addition to the inevitable slaughter, will take no further action until the official despatches detailing the proceedings of the conference have reached England. This is a very different and much wiser course than sending an immediate ultimatum to President Kruger, because, primarily, he will not concede the rights of the franchise to British residents after five years as demanded by Sir Alfred Milner in contradistinction to seven. It may be essential to resort to the threat of force, but at present I am sure that the earnest desire of all sensible persons here and in South Africa is to maintain peace at any price, save dishonour. Happily, the High Commissioner is a man in whom implicit confidence may safely be placed.

The Government should really find time this session to adopt, at least in substance, the admirable suggestions of the Women's Trade Union League, who desire to reduce to a minimum the risks to workers in match factories. I am not an ardent admirer of trade unions, which too often seem to me to be careless how they sacrifice the interests of the community so long as they pursue their own ends. But in this case the remedies put forward coincide with those of the Chief Inspector of Factories. It is proposed to forbid the labour of young people of under eighteen in premises in which yellow phosphorus is used; to insist upon the monthly medical examination of the employés by a qualified surgeon and dentist; special ventilation of the work-rooms; sufficient appliances and time for washing; separate dining and cloak rooms; the restriction of the amount of yellow phosphorus that may be used in the paste; alternative employment for persons engaged in dangerous practices; the appointment of a woman factory inspector; and the extension of the Workmen's Compensation Act to injuries from "phossy jaw." There is a recommendation that the surgeon and dentist should be paid by the State, but though this would ensure freedom from inside influence, the principle is open to objection. All the same, those who know the frightful suffering of workers attacked by "phossy jaw," and the evil effect of yellow phosphorus on the female constitution, will agree that something should be done at once.

The list of those who have obtained distinguished honours at Cambridge this year is singularly representative. The two Senior Wranglers, bracketed as equal, are Mr. G. Birtwistle, an Englishman, and Mr. Raghumath Purnshottam Paranjype, a native of the Murdi District of India. The third on the list is Mr. Samuel Bruce McLaren, an Australian. So our native land, our great dependency, and our colonies have every reason to pat each other on the back, and congratulatory compliments should be flying across the seas and back again. How proud Mrs. Birtwistle must be of her son! His father died eight years ago, leaving a widow with four young children to maintain. At the age of eleven the boy gained a scholarship, and since then, by the winning of prizes and scholarships, he has provided for his own education. First of all England in the Junior Division of the Cambridge Local Examinations, he is now first of all England amongst the Wranglers. Two ladies have also come out splendidly, though not quite equal to last year. Miss M. W. Lapthorn and Miss L. Ashcroft are both Wranglers, one between the 21st and the 24th places, and one equal to the 36th.

I have often heard in marriages amongst the lower classes of the bride having to buy the wedding ring, but according to a case heard in the County Court the other day it appears that quite a trade is done in the hiring of real 22-carat wedding rings. A lady, described as a bottle washer, was sued for £1 3s., which was the amount of hire at 1s. per week due for the wee golden circlet, 9s. having already been paid on it. It does not appear that any portion of the latter sum had been contributed by the husband, though the wife announced her intention of making him pay up some day. The fact that the ring had already found its way to the pawnshop did not speak very well for the future felicity of the married couple. I am certain that if I had to buy my own symbol of matrimony I should consider that a much less expensive ring than one costing 32s. would be quite enough to celebrate my union with so thriftless a husband.

Talking of marrying and giving in marriage, do you see that Paderewski's somewhat mysterious expedition to Warsaw was in reality a wedding journey? He had kept the secret very well apparently, for no one but a few intimate friends knew anything about it. The happy couple have gone to Lausanne for their honeymoon, and I am sure that the thousands of admirers of the talented pianist will wish him every happiness. Fraulein Hélène Rosen is Paderewski's second wife. He has been a widower some years, and he must have needed all the consolation which his music could afford him, for his son is said to be a confirmed invalid.

The idea of "famine prices" in coal sounds absurd in June, does it not? But all the big coal merchants in London anticipate, or appear to anticipate, something of the kind before winter sets in. At any rate, they are sending out alarming circulars calculated to frighten prudent housewives out of their cash, if not out of their senses. One of the largest firms holding a contract to Her Majesty's Government takes "the opportunity" of informing their customers that "without doubt an advance will take place on August 1st." They intimate that the statement "is arrived at by reason of the heavy shipment of coal for abroad, and by rapid and successive advances in miners' wages," and by way of driving the nail home they add, "we strongly advise consumers to lay in their stock before the end of July, and so avoid the possibility of famine prices." Some of my friends tell me that this is all a "trade dodge to secure customers in the dull season," but if so it is very discreditable, and ought to recoil on the heads of those who descend to it.

I have always rather set my face against motor cars in any form, partly because they always seem to me so dangerous, partly because they are so ugly. But on Sunday I saw a new form of motor vehicle rapidly making its way out of town to green fields and pastures new, which almost reconciled me to the modern mode of progression. In a neat little low basket phaeton, resembling very much in appearance the carriages which are to be met with at most seaside resorts, sat side by side a lady and her little child about four years of age. There would hardly have been room for two full grown people, so that I infer the carriage had been designed especially for its occupants. In front, taking the place of the pony, was a gentleman on a motor bicycle, and he was going along most easily, without any exertion, at a great rate, the weight of the basket phaeton which he drew apparently being too insignificant to be taken into much account. Altogether the little family party looked so happy that I nearly envied them, and wished I knew a friend with a similar apparatus who would take me out for a trot.

### Royal British Nurses' Association.

ANNUAL MEETING.

The annual meeting of the Royal British Nurses' Association was held at the Westminster Town Hall on Saturday. The President, H.R.H. Princess Christian, attended, and was presented by Mrs. Coster, the honorary nurse secretary, with a bouquet of orchids. Amongst those present at the meeting were Sir James Crichton Browne, (who at the outset took the chair), Mrs. Dacre Craven, Miss De Pledge, Miss Thorold, Miss Wedgewood, Miss Kelly, Miss Georgina Scott, Miss Pruett, Dr. Outterson Wood, Dr. Bezly Thorne, Mr. Pickering Pick, and the matrons of several important metropolitan and provincial hospitals. After the usual formalities had been observed and the scrutineers elected to examine the voting papers,

Mr. Langton, hon. treasurer, brought forward the financial report up to March 31st. As it had already been printed and circulated in the May number of the *Nurses' Journal*, it was taken as read, and Mr. Langton proceeded at once to explain the rearrangement of the form in which the accounts are presented. The benevolent account is now kept separate, and is for the future to be known as the "Helena" Benevolent Fund; the journal is also placed on a distinct footing. The Treasurer drew especial attention to the fact that for the first time the balance-sheet showed an excess of income over expenditure of £71.

Dr. Bezly Thorne paid a warm tribute to the hon. treasurer's kindness and untiring efforts. He had piloted the Association through many troubles into smooth water. Dr. Thorne also thanked the members of the association for their loyalty to their hon. officers and to their gracious president.

Miss De Pledge emphasised the gratitude with which all the members recognised the hon. treasurer's services. She said she had worked with him as nurse, as colleague, as friend, and time had only deepened the respect and admiration she felt for his character. In her judgment, no thanks could be too good for him.

Mr. Edward Fardon next presented the report of the executive committee. This was also taken as read. Several measures, Mr. Fardon stated, had been organised and initiated during the year which could not fail to produce beneficial results in the future. One was the publication of a complete list of all the nurses whose training is registered by the society, as well as of the nurses who are members of the society. A further record of nurses qualified in any special subject had been added, and this should be of value both to the nurses themselves and to the public. He would urge on all trained nurses the advisability of taking a midwifery qualification as well as their three years' hospital training, in view of the possibility of the measure now before Parliament becoming law. This measure would register women who had acquired a knowledge of one branch of the profession by three months' training, and it was necessary to fully guard the interests of the really trained nurses.

Miss Pruett explained the advantages which had resulted from the establishment of lady consuls in provincial centres. The benefit to colonial and provincial trained nurses had already been considerable.

Princess Christian then took the chair and announced that her sister-in-law, the Duchess of Connaught, had consented to become a vice-president of the Association. She submitted the names of Mrs. Dacre Craven, Sir Richard Thorne Thorne, K.C.B., Sir Richard Douglas Powell, Bart., M.D., and Dr. Bezly Thorne to be vice-presidents, all the names being received with acclamation. Her Royal Highness continued: "I have a further announcement to make of an agreeable character. You know that I have been endeavouring to increase the funds of the Association. I am told it has been said that I have held up nurses and the nursing profession as objects of charity. I am very sorry for the people who made that remark. I think the nurses themselves must have seen how, from the highest to the lowest, everybody came forward to help on a recent interesting occasion, and the result has been that I am now able to hand over to the treasurer the sum of £1,551."

The Chairman, in thanking the Princess for her presence that day, observed that to their Royal President's purpose and determination to help the nurses the association owed its origin and its continuance.

Mrs. Dacre Craven recommended the nurses to follow their president's example in courtesy. She related how a poor mother was loth to allow her child to go into hospital "because the sisters were so brusque." The mother added, "I've been in service in high families, spoken to many great ladies, and even to the Princess Christian herself, and she never spoke to anyone as the sisters did." The nurses present must permit her to remind them "that nursing is not science, nor the acquiring of Latin terms; it is the knowledge of what will make a patient most comfortable and easy put into practice." Let them copy their Royal President, and remember the exhortation written many centuries ago, "Be pitiful; be courteous."

Miss Wedgewood moved and Miss Kelly seconded a vote of thanks to the honorary officers.

At the conclusion the Princess presented the badges of membership to 62 nurses, of whom 23 were lady consuls.

The members subsequently assembled at Earl's Court Exhibition for the usual réunion.

### Factory Girls' Country Holiday Fund.

A meeting on behalf of the Factory Girls' Country Holiday Fund was held last Friday at New College, Oxford, under the chairmanship of Professor Margoliouth. Miss Alice Ravenhill (associate of the Sanitary Institute and lecturer to the National Health Society) was the principal speaker, and gave an interesting address in which she alluded to the development of the factory system, and to England's obligations to the industrial army which had placed her in the forefront of civilisation. Miss Ravenhill spoke of the effects, physical, mental, moral, and economic, of monotonous employment; of the universal "birthright of health"; the responsibilities of common Christianity and citizenship; and the knitting together of classes and the benefits to future generations aided by the work of the fund. The annual report of the committee states that 1,724 girls and women were sent to the country or the sea last year. Lady Dysart lent a farm house for the use of the fund for the whole of the summer, 153 girls being thus enabled to enjoy all the delights of a fortnight's stay in the country. Miss Canney, hon. secretary, will be grateful for any help in this direction, and to hear of places for the girls to go to in August, the only time when it is possible for many of them to have their much needed holiday.

### Appointments.

Edinburgh City Parochial Hospital, Craiglockhart.—On June 5th, Miss Ada Beale was appointed Lady Superintendent. She was trained in the Chichester and the Portsmouth Royal Infirmary. Subsequently, she was for three years at the Chalmers' Hospital.

South Lincolnshire Hospital.—On May 26th Miss F. M. Bates was appointed Matron. She was trained at the Royal Infirmary, Newcastle-on-Tyne, and has been head nurse of Darlington Hospital and Dispensary.

Victoria Children's Hospital, Hull.—Miss E. C. Halliday was elected Matron on May 25th. She was trained, and subsequently held various posts, at Charing Cross Hospital.

### Everybody's Opinion.

[Correspondence on all subjects is invited, but we cannot in any way be responsible for the opinions expressed by our correspondents. No communication can be entertained if the name and address of the correspondent is not given, as a guarantee of good faith but not necessarily for publication, or unless one side of the paper only is written on.]

### POOR LAW NURSING.

"A LOVER OF JUSTICE" writes: Seeing the numerous resignations throughout the workhouse infirmaries, the question naturally arises, Why do so many really good nurses resign their posts? I think it is because the conditions under which they work are such that no nurses of three years' training will stand them. First, there is the food, which is a great item, for it is often of such a character that the nurses cannot eat it; then the hours on duty, with so little recreation and the uncomfortable lodgings. I know an institution where the food is insufficient and by no means of the best quality. The day nurses are expected to be on duty at a quarter to six a.m. and not go off till eight p.m., except two evenings in the week, when they are off duty from six to half-past nine. Once a fortnight on Sunday they again leave the wards from six to half-past nine. The apartments provided are meagrely furnished and far from comfortable.

### RUSSIAN FAMINE RELIEF FUND.

MR. J. T. WOOLRYCH PEROWNE writes from 3, Bryanston Place: I have to thank you, as honorary treasurer of the Russian Famine Relief Fund in England, for your kind reference last week to the distress prevailing in the south-east parts of Russia from the terrible famine and still more terrible diseases of scurvy and typhus resulting from it. As an instance of this notice I have received £5 18s. 6d. from correspondents who mention THE HOSPITAL by name in their letters, and doubtless I owe a still larger sum to the words which you wrote, because it is quite likely that all your readers who answered to the appeal did not mention the paper. I have in all cases where an address was given sent a formal receipt for the money, but I should be glad if you could find room for me to acknowledge the following donations sent anonymously: M. D. B., Kent; J. R., R.B.N.A.; A Hospital Nurse; To Help the Poor Russians; M. L, Harrogate; A Sister at the General Hospital, Bristol. As nine shillings saves a human life your readers who have so generously subscribed will have been the means of saving twenty-eight human lives.

### "DON'T."

"THE WRITER OF 'DON'T'" sends the following: "A Very Old Pro.'s" attack upon "Don't" is really most amusing, because it is so serious. Must one always be taken *au grand serieux?* Anyone with the slightest sense of humour would see at once that some of the abused "Don'ts" were meant to be taken metaphorically only. That there were several truisms, though, as well among them is perfectly obvious to any nurse. For instance, it was at my own training school that the assistant matron—a very young-looking woman—was once mistaken for another probationer by a raw recruit. With regard to the caution not to kneel down when on duty—surely my critic does not take it so literally as to imagine a new nurse should die the death of a martyr rather than do such a thing? But perhaps, as it is so long ago, she has forgotten her own early tendencies to drop on her knees whenever possible; for instance, as a housemaid would when she had to clean furniture, &c., or, again, by the bedside of a patient, especially during a tedious dressing. Did she never fall a victim to this breach of hospital etiquette when she was still only very new? Has she never been reproved by some-one or other in authority for being "so very unprofessional?" As to the spatula and poultice knife, has my critic never yet seen, or even heard of, a glass spatula, which certainly is quite unlike any poultice knife? Yet, as the latter is also a spatula, the confusion often exists. Truth is stranger than fiction, "Very Old Pro." The few home-truths in "Don't" were all founded upon facts. Those who look on generally see most of everything in life, and an ordinary sense of the ridiculous helps to impress episodes upon one's mind, unless one purposely shuts one's eyes. But then it takes some of us a very long time to "grow eyes" at all.

### Central Bureau for the Employment of Women.

THE first annual meeting of the Central Bureau for the Employment of Women was held on Monday afternoon, by kind permission of Lord and Lady Wolverton, at Brassey House, Park Lane. The Earl of DUDLEY, representing Lady Dudley (chairman of committee) presided, and in his opening address said that the special object with which the bureau had been formed was not to take the place of other societies already existing for the purpose of providing employment for women, but to combine the forces of those existing agencies, enabling them to co-operate through some common centre. In the second place, the bureau aimed at becoming more than a mere registry office or centre of organisation. A wider side of its work would consist in the collection and dissemination of information and advice amongst employers and employed. Its recognition in this way as a centre should exercise an important influence on women's employment, and lead to a better regulation of supply and demand. Lord Dudley appealed for funds to carry on the work of inquiry, which necessarily involved considerable expenditure. Mrs. Sidgwick, Principal of Newnham College, spoke of the great help afforded by such a central body, and of the difficulty she often experienced in advising students as to their future careers. Mr. Haldane, Q.C., M.P., made an amusing speech on the "servant problem," confessing that the advent of the "lady help" filled him with consternation. Mr. Tennant, M.P., indicated some of the newer openings for trained women as sanitary inspectors, School Board teachers, and factory inspectors, avowing his conviction that these would increase, and that trained and educated women might do good and useful work as manageresses and forewomen in factories. Miss Pycroft, Organising Secretary of Domestic Economy, L.C.C., gave some valuable and interesting details as to the various directions in which she believed there were openings for women, emphasising the need in every case for special training and preparation. There was a large attendance, which included the Countess of Dudley, Adeline Duchess of Bedford, Sir Robert Giffen, K.C.B. (treasurer), Mr. and Mrs. Asquith, Mrs. Wynford Phillipps, Miss Bateson, and Mrs. Percy Boulnois.

### Presentations.

BROMPTON HOSPITAL.—On the resignation by Miss Davidson of the post of lady superintendent at the Brompton Hospital, the sisters and nurses have presented her with silver brushes and mirror, a scent bottle, and a silver-mounted jar. The nurses belonging to the private staff have given her a travelling clock in silver and morocco case, with suitable inscription and monogram engraved on it; and the servants a very handsome brown leather bag, with brass fittings, as a token of good will and appreciation of her interest in them.

EDINBURGH PAROCHIAL HOSPITAL.—On June 12th Miss Alice J. Smyth, lady superintendent, Edinburgh Parochial Hospital, Craiglockhart, was presented with a beautiful gold watch and pearl brooch from the officials of the institution on the occasion of her leaving for mission work in Africa. Miss Smyth, who has held the offices of nurse and lady superintendent for the past eleven years, was the recipient of numerous other gifts, including a cheque from the members of the Parish Council. She leaves for Africa early in July, under the auspices of the Universities Mission.

### Where to Go.

ROYAL ALBERT HALL.—Wednesday, June 21st, 22nd. Bazaar in aid of the Charing Cross Hospital.

### For Reading to the Sick.

"FOR this is the message that ye heard from the beginning, that we should love one another."

"My little children, let us not love in word, but in deed and in truth."—1 *St. John* iii., 11, 18.

"Love is of God, and every one that loveth is born of God, and knoweth God."

"He that loveth not knoweth not God; for God is love."—1 *St. John* iv., 7, 8.

It is not love received
That maketh man to know the inner life
Of them that love him: his own love bestowed
Shall do it! Love thy Father! and no more
His doings shall be strange! —*J. Ingelow.*

I ask Thee for a thoughtful love,
Through constant watching wise,
To meet the glad with joyful smiles
And wipe the weeping eyes;
And a heart at leisure from itself
To soothe and sympathise.
Wherever in the world I am,
In whatsoe'er estate,
I have a fellowship with hearts
To keep and cultivate,
And a work of lowly love to do
For the Lord on whom I wait.
—*A. L. Waring.*

**Reading.**

Love is born of God, and cannot rest but in God above all created things. He that loveth . . . giveth all for all, and hath all in all. Nothing is sweeter than love, nothing more courageous, nothing higher, nothing wider, nothing more pleasant, nothing fuller nor better in heaven and earth.

Love is a great thing, yea, a great and thorough good. By itself it makes everything that is heavy light; and it bears evenly all that is uneven, for it carries a burden which is no burden, and makes everything that is bitter sweet and tasteful.

The noble love of Jesus impels a man to do great things, and stirs him up to be always longing for what is more perfect.

You must one day be severed from all, whether you will or not.

Keep near to Jesus both in life and in death, and commit yourself to His faithful care, Who, when all others fail, is able alone to help you.

Love Him and keep Him for your friend, and He will stand by you when all other friends depart, and will not suffer you to perish at the last.—*Thomas à Kempis.*

Guided by love, then, let us each look carefully upon our own conscience, and see how far we are loving our dear Lord with all our heart, all our mind, all our soul, and all our strength, not only avoiding great and obvious sin, but in the diligent cultivation of those virtues and graces which are as precious flowers in the Lord's garden, pleasant and acceptable in their fragrance to the Master of the vineyard.—*H. L. Sidney Lear.*

Even so, who loves his Lord aright
No soul of man can worthless find.
All will be precious in his sight,
Since Christ on all hath shined.
But chiefly Christian souls; for they,
Though worn and soiled with sinful clay,
Are yet to eyes that see them true
All glistening with baptismal dew.

So is it with true Christian hearts;
Their mutual share in Jesus' blood
An everlasting bond imparts
Of holiest brotherhood.
Oh! might we all our lineage prove,
Give and forgive, do good and love,
By soft endearments in kind strife,
Lightening the load of daily life! —*Keble.*

### Notes and Queries.

**The contents of the Editor's Letter-box have now reached such unwieldy proportions that it has become necessary to establish a hard and fast rule regarding Answers to Correspondents. In future, all questions requiring replies will continue to be answered in this column without any fee. If an answer is required by letter, a fee of half-a-crown must be enclosed with the note containing the enquiry. We are always pleased to help our numerous correspondents to the fullest extent, and we can trust them to sympathise in the overwhelming amount of writing which makes the new rules a necessity.**

**Every communication must be accompanied by the writer's name and address, otherwise it will receive no attention.**

*Acetic Acid.*

(104) Would you kindly inform me through THE HOSPITAL for what purpose acetic acid is used externally, and oblige?—*A. M.*

Diluted acetic acid is beneficial for certain eruptions and as a hair wash; stronger, it is useful for insect bites and wasp stings; and stronger still, for removing warts. It is also used in cases of ringworm. A weak solution sponged over the body in fevers seems to have the effect of lowering temperature, while hot solutions repeatedly applied over parts affected with rheumatism or neuralgia often give much relief to the pain, so much indeed that certain quacks have advocated its use as a system of cure, which they have ingeniously termed "acetopathy."

*Recognition of Training.*

(105) Would you kindly tell me whether a nurse engaged for a three years' training in a fever hospital can reasonably expect to be trained in all the diseases taken in, or only in such as those in authority care to give? Can they, for example, refuse to give her training in diphtheria or enteric fever if the nurse herself wishes it? I should also be glad if you could give me any information about the hospitals in New York, and whether a "fully trained certificated nurse" would be recognised as such in America, or an American trained nurse's certificate be recognised in Britain.—*Nurse.*

It is very reasonable and right, under the above-mentioned circumstances, that a nurse should be fully trained in nursing all kinds of fevers, but there is no means of compelling authorities to consider the nurse's training and not their own convenience. The certificates granted by the best training schools in America and at home are mutually recognised in both countries, but we warn you that considerable jealousy is felt in America against English nurses, and consequently much difficulty is experienced in establishing a connection.

*Secretaryship.*

(106) My sister is anxious to get an appointment as secretary or assistant secretary in a convalescent home or an institution at Broadstairs or neighbourhood. Would you kindly inform me where she can make inquiries for such?—*Nurse.*

These appointments are difficult to hear of, and are generally filled up privately.

*Three Years' Certificate.*

(107) Could you tell me of any hospitals where I could work for two years (having previously done a year's general work in hospital), and receive a three years' certificate?—*Phyllis.*

The only hospital that professes to do so is the Hospital for Consumption and Diseases of the Chest, Victoria Park, London, E. By consulting the "Nursing Profession: How and Where to Train," you will be able to see which hospitals grant a two years' certificate. Write to the Local Government Board before you make definite arrangements, and ask if they will recognise the one year's certificate that you already hold, and the two years' certificate that you propose acquiring, as "three years training in a recognised training school," otherwise all your trouble would be useless.

*Nursing Abroad.*

(108) I am advised to winter abroad, as I suffer intensely from cold and from weak circulation. Any of the warmest places on the Riviera would do, or Cairo. Kindly tell me how to set about it, and the best time to go. I am anxious to take out an invalid. An article on Las Palmas, Grand Canary, appeared in "Mirror" of February 11th last. Would there be work for a private nurse there?—*Nurse A. A.*

You say nothing of your training. In the event of that being up to the standard required by the Colonial Nursing Association (Imperial Institute, London, W.) you might hear of work through the Secretary. As to taking out an invalid, your best plan would be to advertise to accompany patient. It would not be safe to trust to the chance of private nursing at Las Palmas.

(109) Could you kindly tell me if there is an institution from which nurses are sent abroad for the winter season?—*A. L. S.*

The Holland Institute, 1, Tavistock Chambers, Bloomsbury, W.C. Advertisements of vacancies appear from time to time as they occur in the "Mirror."

*Artificial Leg.*

(110) Please give me (1) the name of a firm in England where artificial limbs can be procured. (2) Can you give some idea as to what the cost of an artificial leg for a man would be? (3) Can second-hand ones be obtained?—*Nurse May.*

This seems a case in which to apply to the Surgical Aid Society, Salisbury Square, Fleet Street, E.C. It exists for the purpose of supplying the really poor with costly appliances, which they could not otherwise obtain. (2) The cheapest legs are from £15 15s. in an ordinary way. (3) In buying a second-hand one, would there not be some difficulty as to the size?

### Travel Notes.

By Our Travelling Correspondent.

### XXVII.—SAN REMO.

Once more we are back on the Riviera, and now we are in Italy; the Italian Riviera is definitely cheaper than the French, and not quite so fashionable; as to its relative beauty and climatic advantages I think there is little difference.

### Expenses of the Journey.

The cheapest way is viâ Newhaven and Dieppe, Aix-les-bains and Turin, first-class, £6 11s. 9d.; second-class, £4 12s. 3d.; the route viâ Newhaven and Dieppe but by Marseilles and Ventimiglia is almost the same, only three shillings dearer. The short sea route viâ Dover and Calais, first-class, £7 19s. 8d.; second-class, £5 9s. 6d. I have so frequently mentioned the best stopping places *en route* that it is unnecessary to repeat them.

### Hotels, Pensions, and Apartments.

The name of the hotels is legion, and, speaking broadly, their charges are moderate. The Hotel Royal is very popular with the English. Pension from 10 to 18 fr., according to rooms; the table d'hote is at separate tables, which our exclusive nation likes. Then the Iles Britanniques is comfortable and has tennis courts. Terms from 11 fr. Considerably cheaper are the Hotel Paradis and Hotel de la Reine; pension from 9 fr. There are also several pensions well recommended; these are all on the west side. On the east, the Bellevue, Victoria, and Mediterranée are all about the same in terms and equally good; pension from 11 or 12 fr. Less expensive are the Europe, the Cosmopolitan, and the Paris; pension from 8 fr. Villas are rather expensive, and, as I have often said before, I think as a rule they are a mistake. The rent for a term from October to May ranges from £80 to £450, but those at £80 are few and far between. Furnished apartments are difficult to meet with. The drainage is good, though on an old-fashioned system, and the supply of water is excellent; this probably accounts for the

### Healthiness of San Remo, on which the inhabitants and appreciative visitors greatly plume themselves, and seemingly with some reason, for it enjoys a distinct immunity from zymotic diseases. I must confess myself to a partiality for San Remo over Nice or even Mentone, it is so gay and bright, without being crowded to suffocation by English and Americans, and is distinctly Italian in character.

### Churches, Shops, Club, Library, &c.

There are four churches, beside the Roman Catholic one of St. Siro, All Saints in the Corso dell' Imperatrice and St. John the Baptist, Via Roma, both Anglican. In the Corso dell' Imperatrice is also the Scotch church, and in the Via Umberto is a French Protestant church which is called l'Eglise Vaudoise. There are plenty of good shops, quite on a par with those of Mentone and Cannes, six English doctors and a staff of trained nurses at 18, Via Roma, two good dentists, an English chemist, and an excellent circulating library at Gandolfo's. Admission to the club is obtainable by a subscription of 12 francs a month. Balls are given in the season. Cabs are much the same here as elsewhere, but carriages for more distant excursions are distinctly cheap, though I made most of my journeys in the humble diligence or on my own legs. If health permits, and you have the true spirit of the explorer and a proper disregard for luxurious travelling, these drives in company with the country folk are very entrancing, especially if you can speak their language even a little; they take you, so to say, to their hearts at once, and help out your stammering efforts with much readiness.

### Old San Remo is interesting and picturesque beyond description. The necessity for strengthening the houses and walls against the shocks of earthquake rendered it necessary to throw connecting archways across all the narrow stairways, which are the only means of communication in the old town. Mules, as usual, are the useful beasts who carry everything, from old women of fearful aspect to loaves and flowers. I give you a sketch of one of these extraordinary stairways with a bewildering number of arches thrown across; notice the little garden high above your head on the right side. If you have the artistic instinct even feebly developed you will enjoy a visit to the old cathedral church

An Old Side Street in San Remo.

of San Siro. It is a matter of considerable difficulty to find it, for the old tunnels, stairways, and bewildering little courts are worse than the maze at Hampton Court; one sees it appear and disappear like a dissolving view through various openings in the tall houses, but ever separated by an impassable gulf; at last after much research patience is rewarded. I saw it for the first time on Good Friday, and recognised the truth of J. A. Symonds' description in his article on the Corniche: "In the cathedral church of San Siro on Good Friday they hang the columns and the windows with black; they cover the pictures and deface the altar; above the High Altar they raise a crucifix, and below they place a catafalque with the effigy of the dead Christ. To this sad symbol they address their prayers and incense, chant their 'litanies and berries,' and clash the rattles, which commemorate their rage against the traitor Judas. . . ." The sun can penetrate only a little way into the mounting passages, but all the same flowers bloom brilliantly and give vivid patches of colour against the frowning houses, black with the weather of many centuries. Shops are few and far between; a few stalls for salt fish and chandlers' wares, and booths for selling the extremely sour wine of the country, is all that meets the eye. The marketing is done in the square below, outside the grim old town. By choosing the hour when young San Remo was threading the thorny road of learning in the various schools, I sketched with great ease, for the adult Italian is ever a gracious and courteous person; indeed the chairs and tables offered were sometimes an embarrassment, but I would not for worlds have declined them. The drives and excursions around, of which I will tell you something next week, are infinite in variety and beauty, and one good thing is that they are all accessible for invalids by carriage, except of course such pilgrimages as to Lampedusa.

### TRAVEL NOTES AND QUERIES.

Rules in Regard to Correspondence for this Section.—All questioners must use a pseudonym for publication, but the communication must also bear the writer's own name and address as well, which will be regarded as confidential. All such communications to be addressed "Travel Editor, 'Nursing Mirror,' 28, Southampton Street, Strand." No charge will be made for inserting and answering questions in the inquiry column, and all will be answered in rotation as space permits. If an answer by letter is required, a stamped and addressed envelope must be enclosed, together with 2s. 6d., which fee will be devoted to the objects of the "Hospital Convalescent Fund." Any inquiries reaching the office after Monday cannot be answered in "The Mirror" of the current week.

Pau (Hermes).—Not a cheap place by any means. Hotel Gassion is the best, with a fine view, but expensive unless you go up high and take a north room. Under those circumstances, if you stay long you can manage it from eight to ten francs per day. Pensions few and expensive. It is quite a ville de luxe, but very delightful.

Central Italy (Artist).—I do not consider that the scenery in Central Italy is particularly interesting in itself, being often very wild and barren, but the neighbourhood of the fortress towns, such as Perugia, Siena, Orvieto, &c., teems with charming views. For pure landscape you would do better in the north among the lakes, or in such neighbourhoods as Sorrento, Amalfi, Castellamare, &c. The scenery round Rome stands alone in its peculiar desolation. The difficulties of doing justice to the scenery of the Campagna are great.

Spanish Frontier (Isabel).—I do not think you will find any difficulty in sketching, always provided that you ask permission of the authorities. I generally applied to the Carabineros, who answer to the French Gendarmes. If you omit this precaution you will speedily find yourself in the nearest lock-up; the same rule applies with even greater force to photography.

Western Brittany (Solo).—Trains in Brittany are very slow; it will take you the entire day to go from St. Malo to Morlaix. If you wish to stop *en route*, I think I should choose Guingamp; there is a fine church and some picturesque corners. From Morlaix to Quimper is another entire day's journey. They are a leisurely people, and do not understand hurry. The alternative way would be to go from Dinan to Vannes. You would have to sleep one night at Ploermel.

Western Scotland (May Flower).—In the tourist season you can have a return ticket to Oban via Glasgow for £3 2s. There are innumerable circular tours published for the West of Scotland and the Isles, but space prevents my putting them in. Write to Messrs. Gaze, 142, Strand, and ask them to send you the list of circular tours in Scotland for the tourist season. Also give them your exact desired route, and ask what the cost would be. You would probably like to make a tour round the Western Isles from Oban. Hotels in Scotland are very dear, especially at that season; you must reckon on 10s. a day, and then you will be doing it very reasonably, and this only covers hotels. Thirty days at 10s. would be £15 each, and your tickets you will be able to estimate roughly when I tell you your return to Oban is £3 2s. At Glasgow go to the Alexandra Hotel, or, if full, to the George Hotels Company. At Oban to the King's Arms Hotel. It would be a good plan for you to take Gaze Hotel Coupons for these hotels, and you know then your expenses will not exceed 10s. When you have decided your route I shall be pleased to help you further if I can.

Climate and Dress in Jerusalem.—The climate is hot naturally and not so agreeable as that of Egypt, but not subject to such severe changes of temperature between the day and night. Clothes should be light woollen underneath, whatever is worn above, which in this case would be cotton I conclude, and there is no reason against such upper garments, but everything in their favour, but woollen combinations must never be laid aside on any account. As to luggage, there is nothing so convenient as a cabin trunk made of wicker and covered with painted canvas. If a supplementary box is required, take one of those expanding Indian reed cases that fit into the lid, secured with a strap. Price from 4s. to 10s.

*(For Travel Advertisements see Page xvi.)*

### Wants and Workers.

Nurse Seaton desires to say, in regard to our intimation in our issue of last week, that she no longer wishes to offer her free services for three weeks in a fever hospital.

## Figures and Tables

**Figure f1:**